# Peripheral Vision Tests in Sports: Training Effects and Reliability of Peripheral Perception Test

**DOI:** 10.3390/ijerph16245001

**Published:** 2019-12-09

**Authors:** Nils Schumacher, Mike Schmidt, Rüdiger Reer, Klaus-Michael Braumann

**Affiliations:** Department Sports and Exercise Medicine, Institute of Human Movement Science, University of Hamburg, 20148 Hamburg, Germany; mike.schmidt@uni-hamburg.de (M.S.); ruediger.reer@uni-hamburg.de (R.R.); klaus-michael.braumann@uni-hamburg.de (K.-M.B.)

**Keywords:** visual perception, neuropsychological tests, athletic performance, cognition, reliability, Vienna test system

## Abstract

Various studies suggest the importance of peripheral vision (PV) in sports. Computer-based test systems provide objective methods to measure PV. Nevertheless, the reliability and training effects are not clarified in detail. The purpose of this investigation was to present a short narrative non-systematic review on computer-based PV tests and to determine the reliability and the training effects of peripheral perception sub-test (PP) of the Vienna test system (VTS) in a test–retest design. N = 21 male athletes aged between 20 and 30 years (*M* = 26.15; *SD* = 3.1) were included. The main outcome parameters were peripheral reaction (PR), PR left (PRL), PR right (PRR), field of vision (FOV), visual angle left (VAL), and visual angle right (VAR). Reliability was assessed using intraclass correlation coefficient (ICC) and Bland–Altman plots. Training effects were determined by students *t*-test. Good reliability was observed in PR, PRL, and PRR. Moderate reliability was found in FOV, VAL, and VAR. Significant improvements between T_0_ and T_1_ were found in PRL with a mean difference of 0.04 s (95% CI [0.00–0.07]) and in PR with a mean difference of 0.02 s (95% CI [0.00–0.05]). For PRR, FOV, VAL, VAR, no significant differences were detected. These results indicate that PP can be applied to asses PV abilities in sports. Future research is needed to clarify the influence of test repetitions on visuomotor learning in PP. Moreover, PV tests should be cross-validated with sport-specific measurements (e.g., on-field and/or ‘virtual reality’ approaches).

## 1. Introduction

The relevance of perceptual–cognitive abilities in sports has been investigated in various studies [1,2,3,4,5]. Visual perception in general [4,6,7,8] and peripheral vision (PV) in particular are discussed to be of special importance in order to perform in various sports [8,9,10,11]. Nevertheless, in the context of the expert-performance approach, the exact role of PV remains in need of clarification. The usage of different outcome parameters (e.g., peripheral response time, retinal sensitivity, visual discrimination, dynamic visual acuity, and static visual acuity) in different measurement devices to investigate PV, further hinders clear interpretation (see Table 1). In sports research, PV is defined as the ability to detect and react to stimuli outside of foveal vision [4].

On the one hand, experts showed superior visual general “hardware” abilities compared to less skilled athletes or novices, e.g., in peripheral response time [8], retinal sensitivity [12], visual discrimination [13], dynamic visual acuity [14], and static visual acuity [15]. On the other hand, no differences between experts and novices concerning visual acuity in youth soccer players [16] and no effects concerning degraded static acuity in golf players [17] could be found (a detailed definition of the above-named abilities can be found in Planer, 1994 [10]). The relationship between general perceptual–cognitive abilities and sports-specific skills is predominantly investigated in the context of the cognitive component skill approach [3]. The cognitive component skill approach has been criticized for not sufficiently taking into account the complexities of the environment that might generate superior expert performance [2]. This is discussed also in regard to the measurement of general percpetual abilities in sports and potential diminished ecological vailidty of test results as predictors for sport specific on-field skills [18].

Nevertheless, numerous studies indicate that in team sports, visual abilities and especially the ability to perceive in the periphery are important for successful performance [4,6,7,8].

Historically, the Goldman perimeter was one of the first devices capable of performing visual field tests in both static and dynamic perimetry [19]. In contrast to dynamic perimetry, static perimetry means presenting stimuli in fixed locations and increasing the stimuli’s intensity until the participant signals perception. Whereas, in dynamic tests, the stimuli move from outside the field boundaries into the presumed field of view. The location in which the stimuli are perceived is considered the boundary of the visual field. The visual field consists of the central visual field and PV. The central visual field is defined as the field of view with 60° diameter horizontally. The PV then occurs within the area from 60° up to nearly 180° horizontal diameter [20].

Today, devices used to capture PV parameters are mainly automated or computer-based. These systems provide objective methods, as they instruct the subjects in a standardized manner and independently of the test administrator [21].

In sports research, these computer-based methods operationalizing PV are increasingly applied in order to gain insides into relevant cognitive abilities in athletes and differentiating between experts and novices. Consequently, there are many computer-based tests on the market. In the first section of this article, we provide a narrative overview of existing PV test systems. Furthermore, the issue of potential training effects and thus, diminished test–retest reliability, is discussed. In the second section of this article, the test–retest reliability and potential training effects of the peripheral perception sub-test (PP) of the Vienna test system (VTS) is assessed in a test–retest design. The Vienna test system (VTS) is one of the predominantly used computer-based tests in sports science as it operationalizes many sport psychology-related constructs such as sustained attention, stress reactivity, as well as the hereby discussed peripheral perception [21].

### 1.1. Peripheral Vision Tests in Sports

Table 1 shows computer-based/automated tests often used for PV measurements in sports research and respectively, their sub-tests. PV parameters measured are peripheral eye–hand response time, peripheral response time, retinal sensitivity within the visual field, visual processing speed, peripheral vision, peripheral reaction (PR), and visual field (FOV).

In five out of the six presented systems, test subjects have to detect a light stimulus positioned at various points within the field of view while maintaining fixation on a reference point in the central visual field.

The ability measured with these test systems can be labeled as central–peripheral awareness. Central–peripheral awareness is defined as the athlete’s ability to maintain central fixation on a target and be aware of what happens in the peripheral visual field [10]. This function of visual perception evaluates the athlete’s ability to respond to central and peripheral stimuli without moving the head. The majority of these tests use binocular methods (Eye–hand coordination test (EHCT), Wayne computerized saccadic fixator (WCSF), Useful field of view (UFOV), Athlevision (Asics Corporation, Japan)/peripheral vision test (PVT), Peripheral perception (PP)), which means both eyes are used at the same time. The Humphrey field analyzer 630 (HFA) is a monocular test in which the eyes are tested separately. The following describes the procedure, the reliability, and training effects of the above-named PV tests. Furthermore, a short overview of existing studies using the PV test-systems in sports research is presented. The reliability reflects the extent to which measurements can be replicated by an instrument on the same subject under the same conditions [22]. The training effect is understood as the quantity of variation measured between test and retest (without any intervention of participants between test and retest). The assessment of the reliability, and subsequently the training effects in Table 1, is based on the information provided by the cited authors.

#### 1.1.1. Eye–Hand Coordination Test (EHCT) of Nike Sensory Station (NSS)

The participant’s task is to react to a green dot which appear on a touch-sensitive display (42-inch). The display consists of a grid of equally spaced circles (8 columns, 6 rows). In total, 96 dots have to be reached. The participants are positioned at a distance of about one arm length away from the display. The faster the participants work, the shorter the peripheral eye–hand response time, which is measured in seconds (sec).

In sports research, the NSS was used in high school football [24] in pitchers and hitters [23] and in collegiate ice hockey [13].

Test–retest reliability on EHCT was investigated in 125 participants aged between 18 and 30 years via two measurements within one week. A moderate reliability with an average difference of −2.70 s (−8.04, 2.99) between test (T_0_) and retest (T_1_) was found. Thus, the analysis of training effects revealed a significant improvement between T_0_ (*M* = 49.7, *SD* = 4.66) and T_1_ (*M* = 47.0, *SD* = 4.58) (t (159) = 11.76, *p* < 0.001) [26]. Krasich et al. [27] observed in a later study a similar significant training effect.

#### 1.1.2. Wayne Computerized Saccadic Fixator (WCSF) 

The PV outcome parameter in this test [28,29] is the peripheral response time measured in correct responses. The participants are positioned 60 cm away from the central cylinder with 8 peripheral lights mounted on 50 cm long rods in cardinal and ordinal directions. For the procedure, participants are required to press a randomly lit peripheral button with one hand while concentrating on a central stimulus.

In sports research, WCSF was used to analyze general athletes [30,31] and soccer athletes [32].

In an investigation of retest reliability of WCSF, 20 male members of a major league soccer team, aged between 17 years and 35 years, participated in two consecutive trials. The analysis revealed poor reliability (ICC = 0.321, *p* = 0.168) and significant training effects (*p* = 0.007) [33].

#### 1.1.3. Humphrey Field Analyzer 630 (HFA)

The HFA is used to measure the retinal sensitivity within the visual field [31]. The sensitivity is given in decibel (dB). The method is generally accepted as the “clinical standard” to detect and to quantify optical diseases (e.g., glaucoma) [44]. In the static condition, participants have to fixate a central target (monocular) while stimuli are presented randomly at 24 eccentricities along the horizontal meridian. The stimuli are presented in 6° steps from 0° to 72° horizontally in the left and the right visual field. In the kinetic condition, the stimuli are given along 12 meridians and moved with velocity of 0.07 rad*s- 1 centrally until they were detected.

In sports research, the analysis of retinal sensitivity within the visual field measured by the HFA was undertaken in general athletes who had no specific competitive experience [30,31].

In an analysis of training effects, Heijl et al. [34] determined perimetric experience in 210 individuals between 20 and 80 years of age. Ten tests were conducted within one week. Significant improvements between test 1 and test 5/6 were found (average increment 1.3 dB (*p* < 0.001)). The learning was more pronounced peripherally than paracentrally. To our knowledge, there are no reliability studies investigating test–retest reliability on HFA.

#### 1.1.4. Second Sub-Test of Useful Field of View Test (UFOV) 

In UFOV [35], the examinee has to identify a central target, but also localize a simultaneously presented target displayed in the periphery of the computer monitor. The visual processing speed is given in milliseconds (ms).

Appelbaum et al. [36] tested peripheral task performance in football players and general athletes.

The test–retest reliability of the touch PC version of UFOV was investigated by Edwards et al. [37]. Sixty-six participants were included in two measurements within one week. A high correlation between test and retest was observed for the touch PC version (*r* = 0.74) and the mouse PC version (*r* = 0.88).

#### 1.1.5. Peripheral Vision Test (PVT) of Athlevision

The PVT measures the central–peripheral awareness which is given in correct responses. The participants’ task is to find peripheral O’s while watching a central number on a computer display. The O’s are orders peripherally around the central number. The test complexity is increasing. The higher the rank, the further the O’s appear from the center [38].

Using PVT, Chang et al. [39] compared sports vision in soft tennis adolescence athletes. 

Analysis of reliability revealed a high test–retest correlation (ICC = 0.90) [39]. The study of reliability was conducted with 26 elementary school students who took measures (twice at the same time) on two consecutive days. To our knowledge, further analysis on training effects were not published.

### 1.2. Peripheral Perception Test (PP) of the Vienna Test System (VTS) 

The VTS is a computerized test system for psychological assessments in traffic psychology, psychology aviation, human resource (HR), neuropsychology, and sports research [45]. Besides a scientific use of the different constructs of the test, applied practitioners use the different sub tests of the test system in the field of sports science in order to assess their athletes. In sports, the test enables to analyze the personality and the ability of athletes with sub tests of many different constructs such as reactive stress tolerance, choice decision making, motor speed and reaction speed, coordination, sustained attention, and peripheral perception [46].

**Figure 1:**
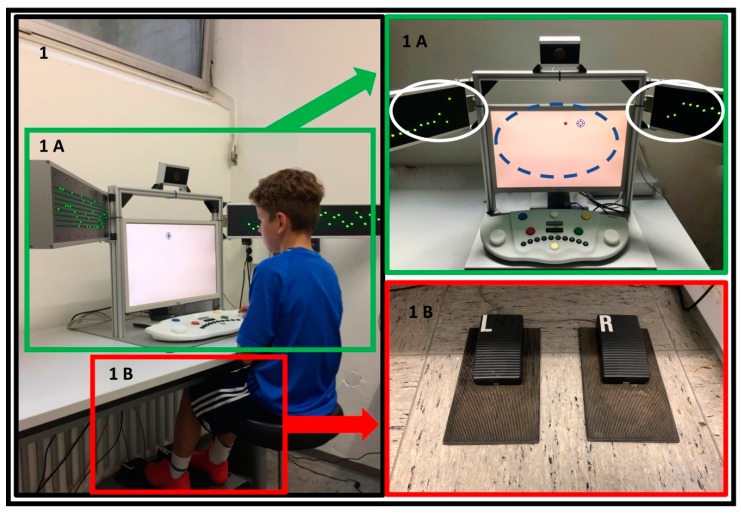
Peripheral perception test (PP) with proband panel (1A, white circle, continuous line) tracking task (1A, blue circle, dashed line) and foot pedal (1B, for left and right foot).

Consequently, the PP subtest (see Figure 1) [45] is commonly used in sports research (see Table 1) [8,11,41,42]. Participants have to simultaneously solve a central tracking task (see Figure 1: 1A, blue circle) and a peripheral perception task (see Figure 1: 1A, white circle). The reaction to peripheral stimuli is given by depressing a foot pedal (see Figure 1: 1B). Main outcome parameters are peripheral reaction (PR) in seconds (sec) and visual field (FOV) in degrees (n°). For a detailed description see section Materials.

Jiménez-Pavón et al. [41] investigated PV in a running exercise. In other studies, peripheral response times were investigated in handball players [8,42] and female basketball players [43].

Although PP was used in sports research frequently, analyses on reliability were not found (as seen in Table 1). In order to further use PP in sports research, knowledge about test–retest reliability is crucial. An adequate evaluation of training interventions (e.g., perceptual–cognitive training) requires a consideration of the reliability and training effects of PP sub-test of VTS.

In summary, computer-based PV measurements are frequently used in sports research investigating general [25,30,31,41], baseball [23], football [24,36], ice hockey [13], soft tennis [39], handball [8,42], and volleyball [11] athletes. The described test systems provide objective methods to analyze PV parameters. Nevertheless, investigations on reproducibility yielded mixed results which indicate that repeated processing might lead to training effects [26,27,33,34]. Only for NSS and WCSF, detailed information on reliability and training effects was found. However, precise descriptions on differences between test and retest are of special importance when assessing the usage of a test system. Training effects might be the possible confounder of the measurements and therefore should be assessed and reported in sports science. This is of special relevance in intervention trials. Especially in regard to the widely used VTS in general, and an increasing use of PP subtest in particular, there is a lack of test–retest reliability studies (see Table 1).

Based on preliminary evidence indicating test–retest improvements on motor-dependent tasks of computer-based PV tests [26,27,33] and because of the emerging popularity of the VTS in sports science and applied sports, in the present reliability study, we aimed to quantify the training effect potentially relevant in practical usage of PP in young, healthy male athletes.

The research objectives of the present study consequently were:

1. to investigate the test–retest reliability and
2. to examine the training effects between test and retest measured with peripheral perception test (PP) of the Vienna test system (VTS) [45].

We hypothesized that peripheral reaction times and field of vision measured with PP sub-test of VTS highly correlate between test and retest. Moreover, we hypothesize significant training effects in motor-dependent tasks.

## 2. Materials and Methods

### 2.1. Study Design

To investigate the test–retest reliability and training effects, measurement was conducted twice within one week (seven days between T_0_ and T_1_) at the University of Hamburg, Department of Sports and Exercise Medicine. Measurements were conducted at the same time of the day on test (T_0_) and retest (T_1_). In terms of better comparability, measurement time was scheduled between 10:00 a.m. and 6:00 p.m. for all participants. Prior to the test, participants had to provide information on their daily routine in a self-constructed diary time table of the day of measurement. Participants were excluded if large changes were detected in daily routine (altered perceived cognitive and/or physiological load). Each participant was instructed via a computer in a standardized manner. The head had to be positioned at a distance between 30 cm and 60 cm from the screen. If the distance was incorrect, participants were respectively requested to sit further away or closer. Moreover, the sitting position was standardized in regard to T_0_ and T_1_ and checked by the examiner. The total time spent on PP testing was about 15 min and each measurement were conducted in a quiet room and under same humidity, temperature, and light conditions.

Ethical approval for the present study was obtained from the local ethic committee of the faculty of Psychology and Human Movement Science, University of Hamburg (AZ 2017_106). Data collection took place anonymously and all measurements were noninvasive. Prior to the study, all participants gave their written informed consent. The study followed the principles of the Helsinki Declaration.

### 2.2. Participants

Considering findings on age and sex-related influence on visual reaction time [47,48], we solely recruited male athletes (*N* = 21) aged between 20 and 30 years (*M* = 26.15; *SD* = 3.10) to ensure a homogeneous population. The participants were recruited from the Institute of Human Movement Science, University of Hamburg. This experiment was part of a larger study on perceptual–cognitive abilities in male soccer players. In regard to potential associations of experience in sport games with visual perception abilities, the participants needed practical experience in sport games for at least 3 years. The subjects stated that they had a similar daily routine at T_0_ and T_1_. They had no experience with computer-based PV measurements, no mental disorders, and/ or other chronic disorders or conditions of the eye. Participants who did not meet these criteria were excluded.

### 2.3. Outcome Measures

PV was assessed using PP sub-test of VTS by Schufried GmbH [45]. The computer-based test consists of two sub tasks, a central tracking task (see Figure 1: 1A, blue circle), and a peripheral perception task. The peripheral perception task is represented on two peripheral panels consisting of vertical and horizontal rows of light diodes which are attached to the left and the right side of the computer monitor (see Figure 1: 1A, white circles). One panel consists of 64 vertical diode columns and 8 horizontal diode rows. Stimuli are given by green lights traveling from the center of the visual field to periphery. Vertical blinking bars (blinking speed: 60 milliseconds (ms)) on the peripheral display are determined as critical stimulus (CS). Participants must react to these stimuli by depressing a foot pedal (see Figure 1: 1B). Time from appearance of CS to pedal pressure is measured in seconds (sec) and defined as peripheral reaction (PR). The tracking tasks to be completed simultaneously are presented on the monitor. Each time the pedal is pressed, an ultrasound distance sensor measures the distance between the monitor and the subjects face in centimeter (cm). These are used for calculation of field of vision (FOV). The CS presentation depends on an adaptive algorithm. Exact diode position could be between position “1” (farthest in) and “64” (farthest out). First CS is always at diode position “33”. When correctly answered, the stimulus moves further into the periphery. If the response is incorrect or the stimulus is not perceived, the stimulus continues to move towards the fovea.

Primary outcomes are PR (PRR and PRL) and FOV (VAR and VAL). PR is calculated by the median of peripheral reaction times for correct responses on right and left CS in seconds (sec). PR is subdivided in peripheral reaction right (PRR) and peripheral reaction left (PRL).

FOV is calculated by visual angle left (VAL), visual angle right (VAR) and is given in degrees (n°). Secondary outcomes are number of hits left (NOHL), number of hits right (NOHR), and tracking deviation (TD). NOH is defined as the number of correct responses to CS (cross-hair in range and foot pedal pressed in response to CS). Due to the adapted stimulus output, subjects with less than 18 hits were excluded. TD is defined as deviation of cross-hair from the target for tracking task in pixel. Therefore, deviation at the moment of every CS (overall 40 CS) is given as a mean.

### 2.4. Data Analysis

Reliability was assessed calculating intraclass correlation coefficient (ICC) [49] for all the six variables (peripheral reaction (PR), peripheral reaction left (PRL), peripheral reaction right (PRR), field of vision (FOV), visual angle left (VAL), visual angle right (VAR)), and the control variables tracking deviation (TD), number of hits left (NOHL), and number of hits right (NOHR). According to previous studies [22], we selected a two-way mixed model with average measurements and absolute agreement. ICC values less than 0.5 was indicative of poor reliability, values between 0.5 and 0.75 indicated moderate reliability, values between 0.75 and 0.9 indicated good reliability, and values greater than 0.90 indicated excellent reliability [50]. The alpha level was established at *p* < 0.05. Moreover, reliability was assessed using Bland–Altman analysis [51]. Therefore, differences between paired variables (test and retest) were calculated and intervals in which 95% of the differences fall (1.96 *SD*, −1.96 *SD*) are given. The distribution of the differences was examined for each variable. The differences were plotted against the mean of T_0_ and T_1_. The average of the two tests (T_0_ and T_1_) is plotted along the horizontal axis. The differences between T_0_ and T_1_ are plotted along the vertical axis.

## 3. Results

### 3.1. Reliability

Reliability was assessed using ICC and Bland–Altman plots. ICC calculation with 95% CI for all variables is shown in Table 2. Good reliability was found on reaction variables RT (ICC = 0.85, 95% CI [0.64–0.94]), PRL (ICC = 0.77, 95% CI [0.41–0.91]), and PRR (ICC = 0.79, 95% CI [0.50–0.92]). Results also revealed good reliability on control variables TD (ICC = 0.82, 95% CI [0.55–0.93]), NOHL (ICC = 0.79, 95% CI [0.47–0.91]), and NOHR (ICC = 0.82, 95% CI [0.57–0.93]).

**Figure 2:**
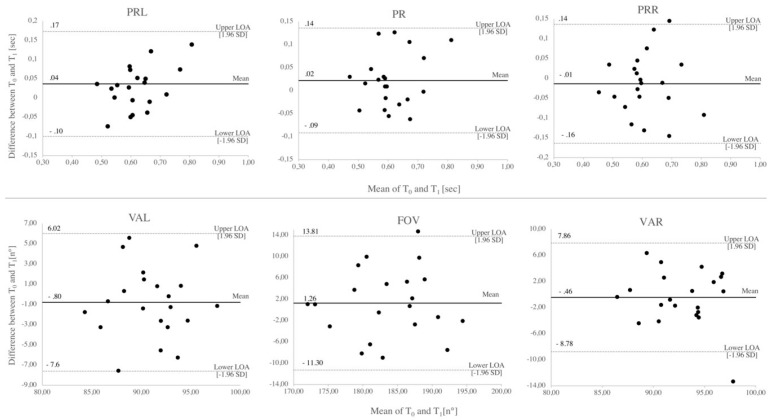
Bland–Altman plots for peripheral reaction and visual field outcomes. Shown are the means of T_0_ and T_1_ on the x-axis and the differences between T_0_ and T_1_ on the y-axis. Values are presented in seconds [sec] and degree [n°]. Dashed lines indicate upper and lower limits of agreement (LOA). Solid lines indicate the mean differences between T_0_ and T_1_. *SD* = standard deviation, PRL = peripheral reaction left, PR = peripheral reaction, PRR = peripheral reaction right, VAL = visual angle left, FOV = field of vision, VAR = visual angle right.

ICC calculation demonstrated moderate reliability on FOV (ICC = 0.73, 95% CI [0.33–0.89]), VAL (ICC = 0.74, 95% CI [0.36–0.89]) and VAR (ICC = 0.58, 95% CI [0.06–0.83]). Bland–Altman plots for peripheral reaction (PR, PRL, PRR) and visual field (FOV, VAL, VAR) outcomes are presented in Figure 2.

For PR, the average difference (LOA) was 0.02 (−0.09, 0.14) seconds. The average difference was significantly different from zero (as described above). For PRL, the average difference (LOA) was 0.04 (−0.10, 0.17). As shown in Table 3, the mean difference was significant from zero. Mean differences for PRR −0.01 (−0.16, 0.14), FOV 1.26 (−11.30, 13.81), VAL −0.80 (−7.6, 6.02), and VAR −0.5 (−8.8, 7.9) were not significant from zero.

Outliers were detected for PRR, FOV, and VAL. On other outcomes (PRL, PR, VAL, VAR) all values were between upper and lower limits of agreement (LOA). The data suggests that the differences on PRL are greater with the highest maximal reaction time. VAR values are slightly shifted above the mean of differences between T_0_ and T_1_. For other plots, no specifics for distribution were detected.

### 3.2. Training Effects

Descriptives for variables T_0_, T_1_, and paired sample t-test for variables are shown in Table 3. There was a statistically significant difference for reaction times of PR between T_0_ and T_1_, with mean reaction times 0.02 s (95% CI [0.00–0.05]) lower at T_1_ (t(20) = 1.73, *p* = 0.01). Also, significant group differences between T_0_ and T_1_ were found for PRL (t(20) = 2.40, *p* = 0.03). The mean difference was 0.04 (95% CI [0.00–0.07]). For other variables (PRR, FOV, VAL, VAR, TD, NOHL, NOHR), no significant differences between T_0_ and T_1_ could be detected. Results for each variable are shown in detail in Table 3.

## 4. Discussion 

The purpose of this study was to determine the test–retest reliability and training effects of PV performance measured with the sub-test PP of VTS in healthy male athletes. Moreover, a short overview on PV test systems and the reliability of test systems applied in sports research was given.

The PP sub-test analysis revealed good reliability in reaction variables (PR, PRL, PRR) and in control variables (TD, NOHL, NOHR). Moderate reliability was found in field of vision variables (FOV, VAL, VAR). Analyses of the training effects showed significant improvements between T_0_ and T_1_ for PRL (t(20) = 2.40, *p* = 0.03) with a mean difference of 0.04 s (95% CI [0.00–0.07]) and for PR (t(20) = 1.73, *p* = 0.01) with mean difference of 0.02 s (95% CI [0.00–0.05]). For other variables (PRR, FOV, VAL, VAR, TD, NOHL, NOHR) no significant differences were found between test and retest.

The most remarkable result to emerge from our analysis is that good reliability was found for PR, PRL, and PRR. Nevertheless, the same parameters (PRL, PR) showed significant training effects. This emphasizes the relevance of additional analyses of training effects in order to evaluate measurement systems. The found training effects on PR and PRL should be considered when using PP as a PV measurement system.

The shown improvements across sessions in PRL and PR might be caused by measured motor response characteristics. These findings support our hypothesis that significant training effects for motor-dependent tasks will be found. Furthermore, this is in line with previous results of computer-based PV measurements (NSS [26], WCSF [33], simple eye–hand reaction time [52]), suggesting improved test results due to enhanced motor responses. Interestingly, in PRR, no significant difference was found between test and retest. This result might be in correspondence to the laterality of measured athletes. Earlier studies showed that handedness, footedness, or eye dominance have an influence on visual reaction times [53,54,55]. We suggest that potential associations between measurement performance, the footedness, and the side the stimuli are presented should be examined. Therefore, it might be of interest to what extent participants responding with the left foot on right sided stimuli react as fast as participants reacting with the right foot on right sided stimuli and vice versa.

Moreover, training effects might be reduced at higher visuomotor skill levels. We found a better and therefore lower mean reaction time right sided at T_0_ and no significant improvement on PRR at T_1_. Whereas, mean PRL was higher at T_0_ and a significant improvement at T_1_ was found. Similarly to training effects found for test repetitions in perceptual–cognitive tests [56], our findings suggest that higher motor and visual skill levels might lead to a “saturation” in visual reaction time. This explanation might be supported by Figure 2, which showed that the differences on PRL are greater with the highest maximal reaction time. However, we did not examine the laterality in our experiment specifically and it is questionable whether these differences are of practical relevance. Therefore, we suggest that future studies using PP sub-test should investigate the influence of laterality (handedness, footedness, or dominant eye) and visuomotor skills on peripheral reaction time.

Further, we found no significant improvements between test and retest in FOV, TD, and NOH parameters. These findings might be consistent with previous ideas, which suggested that processes related to the “visual hardware” are limited by the physical optometric properties of the visual hardware system [57] and thus may not differ between test and retest. Moreover, this could be in agreement with studies, which found no differences between athletes of different expert levels on dynamic visual [16] acuity and static visual acuity [17]. However, also for test systems with less motor demands, as seen at HFA, significant learning effects were observed [34]. Therefore, additional research is required to examine training effects in PV parameters and investigate the neuroanatomical mechanisms underlying improved PV in repeated measurements. It could be helpful to also determine physiological parameters (e.g., cortical blood oxygen saturation) in order to better understand the underlying mechanisms and to consider the resources used for solving dual tasks (tracking task and peripheral perception task). This would then allow to identify motor and cognitive activation in PV measurements in correspondence with activation levels of different cortex areas. Moreover, because of existing findings on a decrease of learning effects with repeated measurements [34], further research should examine how many repetitions are needed to reduce effects of motor learning when using PP and whether behavioral improvements might stem from visuomotor skill learning or from greater familiarity with the testing context and apparatus.

As hypothesized, our analyses of reliability revealed good results in reaction variables (ICC_PR_ = 0.85, ICC_PRL_ = 0.77, ICC_PRR_ = 0.79) and in control variables (ICC_TD_ = 0.82, ICC_NOHL_ = 0.79, ICC_NOHR_ = 0.82). These results are in correspondence with correlations found for UFOV (PC Version: *r* = 0.74/of Mouse PC Version: *r* = 0.88) [37] and Athlevision (ICC = 0.90) [39]. As shown for peripheral eye–hand response of EHCT, we found moderate reliability in FOV parameters (ICC_FOV_ = 0.73, ICC_VAL_ = 0.74, ICC_VAR_ = 0.58). In comparison, for WCSF, only poor reliability was shown [33]. Although moderate reliability was found for FOV variables, the scattering of the measurement values shown in Figure 2 are high and therefore might not be precise enough to detect improvements caused by PV interventions in sports research.

Compared to other computer-based PV measurements, our results of reliability analyses emphasize that PP might be of good test quality. Because of poor reporting on study methods, the quality of some studies analyzing reliability (Table 1) was questionable. These studies especially lack adequate description on participants, procedure [39], and type of ICC [33,39]. However, other studies shown in Table 1 seem to be of good methodological quality [26,27,34,37].

Nevertheless, the present study only showed a broad overview of existing PV measurement systems in sports research and did not aim to systematically review study quality in detail. A systematic review remains to be conducted. Furthermore, we only included young, healthy male athletes with at least three years of experience in sport games such as soccer. With regard to previous studies highlighting gender differences in visual reaction time [47,48,58], these results on potential training effects and reliability of the PP in VTS cannot be generalized with female athletes. This should be considered in future studies. With this reliability study being part of a larger investigation on perceptual–cognitive abilities in game sports, in this study we could only obtain a sample size of N = 21 athletes. Even though study results seem in line with similar studies on different PP measurement systems, results should be interpreted with caution. A priori sample-size calculation (ICC sample size calculation package in R, *p* = 0, p0 = 0.3, k = 2, alpha = 0.05, tails = 2, power = 0.80) shows that in future studies a sample size of N = 83 should be aimed for.

Moreover, with regard to the ongoing debate on the cognitive component skills approach it might be of interest to link on-field performance and other real-world situations with perceptual–cognitive measures. Therefore, a cross validation with more sport-specific measurements and new technology approaches should be considered. For example, it was already shown, that better performance on dynamic visual acuity and visuomotor control measured by NSS accounted for nearly 70 % of the variability in goals scored in a sample of collegiate hockey players [13]. Further, Zwierko et al. [8] used PP to demonstrate a superior peripheral response times in handball players compared to nonathletes. Furthermore, as recommended by Vignais et al. [59], the analysis of visual information uptake in sports might be more predictive by using a ‘virtual reality’-based methodology. In regard to enhanced technological possibilities, some approaches are already able to detect eye movements in ‘virtual reality’. Such approaches promise a high ecological validity—despite a high standardization—and thus should be utilized in future perceptual-cognitive research approaches. The detection of eye movements should further be used to differentiate gaze behaviors and their different functionalities [60].

## 5. Conclusions

Based on our results, future research should consider potential confounding effects of measurement experience on PV. Moreover, due to a higher ecological validity and higher probability to predict sport-specific skills, binocular instead of monocular measurements should be used to asses PV abilities in sports research. This could address measurements of general perceptual abilities (e.g., NSS, VTS) or sport specific measurements in virtual or real situations. In future studies, normative performance data on specific age levels, gender, skill levels, and specific sport types should be collected.

In summary, the present study delivers several findings on the test–retest reliability and training effects of PV performance measures utilized in sport research. Our hypotheses regarding high correlations between test and retest in peripheral reaction times and field of vision detected by the PP test label of VTS and significant training effects for motor-dependent tasks are confirmed by the following results:

- (1) We found good reliability in reaction variables (PR, PRL, PRR) and in control variables (TD, NOHL, NOHR). Moderate reliability was demonstrated in field of vision variables (FOV, VAL, VAR).
- (2) The analysis of the training effects showed significant improvements between T_0_ and T_1_ for PRL with a mean difference of 0.04 s (95% CI [0.00–0.07]) and for PR with mean difference of 0.02 s (95% CI [0.00–0.05]).
- (3) No significant differences between test and retest were observed in the following variables: PRR, FOV, VAL, VAR, TD, NOHL, NOHR.

These results indicate high test quality and suggest that PP sub-test of VTS can be utilized to asses PV skills in sports research. We suggest that future research with larger sample sizes is needed to clarify the influence of test repetition on visuomotor learning in PP sub-test. Further, in order to better understand the underlying mechanisms and to consider the recourse used for solving dual tasks, physiological parameters (e.g., cortical blood oxygen saturation) and eye-tracking methods should be considered.

## Figures and Tables

**Table 1:** PV Tests used in sports research including their test–retest reliability and training effects.

Test/Sub-Test	Procedure/Method	Parameter (unit)	Sports	Reliability	Training Effects
Nike sensory station (NSS)/ Eye–hand coordination test (EHCT)	Subjects have to react with their hands to a light stimulus on a display. /binocular	Peripheral eye–hand response (sec)	Baseball [23] High School Football [24] Healthy young adults [25] Collegiate ice hockey [13]	⟹ [26]	↑ [26]/[27]
Wayne computerized saccadic fixator (WCSF) [28,29]	Athletes are required to react to light stimulus with one hand while concentration on a central cylinder stimulus. /binocular	Peripheral response time (correct responses)	General [30] General [31] Soccer [32]	⇓ [33]	↑ [33]
Humphrey field analyzer 630 (HFA)	*Static condition:* Participants have to fixate central while stimuli are presented. *Kinetic condition:* Stimuli are given along 12 meridians and moved centrally. /monocular	Retinal sensitivity within the visual field (dB)	General [30] General [31]	n.a.	↑ [34]
Useful field of view (UFOV)/ Second sub-test [35]	Examinees identifies a central target while localizing a stimulus in the periphery. /binocular	Visual processing speed (ms)	Football and General [36]	⇑ [37]	n.a.
Athlevision (Asics Corporation, Japan)/ Peripheral vision test (PVT) [38]	Participants have to detect peripheral O’s while watching a central number. /binocular	Peripheral vision (correct responses)	Soft Tennis [39]	⇑⇑ [39]	n.a.
Vienna test system (VTS)/ Peripheral perception (PP) [40]	Subjects have central tracking task and a peripheral perception task need to be resolved (for detailed description see Materials). /binocular	Peripheral reaction time (sec), visual field (n°)	Physical active man [41] Handball [8] Handball [42] Volleyball [11] Basketball [43]	n.a.	n.a.

*Note:* LOA = Limits of agreement. T_0_ = test. T_1_ = retest. Values shown are the mean (M), standard deviation (SD), and 95% confidence interval (CI 95%). Results of pared sample *t*-test (t) are presented with p values and degrees of freedom. (sec) = measured in seconds. (n°) = measured in degrees. (pixel) = measured in pixels. (N) = measured in numbers of hits. (dB) = decibel. Following rating is in accordance with reports by the stated authors: ↑ = significant training effect. • = without training effect. ⇓ = poor reliability. ⟹ = moderate reliability. ⇑ = good reliability. ⇑⇑ = excellent reliability. n.a. = not available.

**Table 2:** Intraclass correlation coefficient (ICC) and 95% CI for variables.

Variables	ICC	95% CI
PR	0.85	[0.64–0.94]
PRL	0.77	[0.41–0.91]
PRR	0.79	[0.50–0.92]
FOV	0.73	[0.33–0.89]
VAL	0.74	[0.36–0.89]
VAR	0.58	[0.06–0.83]
TD	0.82	[0.55–0.93]
NOHL	0.79	[0.47–0.91]
NOHR	0.82	[0.57–0.93]

*Note:* Values shown are the correlations for variables between T_0_ and T_1_ calculated by ICC (two-way mixed effects, absolute agreement, average measurements) and 95% confidence interval (95% CI). PR = peripheral reaction. PRL = peripheral reaction left. PRR = peripheral reaction right. FOV = field of vision. VAL = visual angle left. VAR = visual angle right. TD = tracking deviation. NOHL = numbers of hits left. NOHR = numbers of hits right.

**Table 3:** Descriptives and *t*-test on variables.

Variables	T_0_ *M* (*SD*)	T_1_ *M* (*SD*)	Mean dif. *M* (*SD*) 95% CI	T-Test
PR (sec)	0.62 (0.09)	0.60 (0.08)	0.02 (0.06) [0.00–0.05]	t(20) = 1.73, *p* = 0.01
PRL (sec)	0.64 (0.10)	0.60 (0.07)	0.04 (0.07) [0.00–0.07]	t(20) = 2.40, *p* = 0.03
PRR (sec)	0.62 (0.09)	0.60 (0.09)	0.01 (0.08) [−0.02–0.05]	t(20) = 0.82, *p* = 0.42
FOV (n°)	184.34 (6.98)	183.09 (6.80)	1.26 (6.41) [−1.66–4.17]	t(20) = 0.90, *p* = 0.38
VAL (n°)	91.34 (3.80)	90.54 (3.83)	0.80 (3.48) [−0.79–2.38]	t(20) = 1.05, *p* = 0.31
VAR (n°)	93.00 (4.17)	92.54 (3.54)	0.46 (4.25) [−1.47–2.39]	t(20) = 0.49, *p* = 0.63
TD (pixel)	8.29 (0.87)	8.23 (0.95)	−0.03 (0.72) [−0.36–0.30]	t(20) = −0.18, *p* = 0.86
NOHL (N)	14.33 (3.34)	13.95 (3.4)	0.38 (2.85) [−0.92–1.68]	t(20) = 0.61, *p* = 0.55
NOHR (N)	16.43 (3.27)	15.67 (3.34)	0.76 (2.51) [−0.38–1.90]	t(20) = 1.39, *p* = 0.18

*Note:* Values shown are the mean (*M*), standard deviation (*SD*) and 95 % confidence interval (95% CI). Results of pared sample *t*-test (t) are presented with p values and degrees of freedom. T_0_ = test. T_1_ = retest. Mean dif. = mean differences. PR = peripheral reaction. PRL = peripheral reaction left. PRR = peripheral reaction right. FOV = field of vision. VAL = visual angle left. VAR = visual angle right. TD = tracking deviation. NOHL = numbers of hits left. NOHR = numbers of hits right. (sec) = measured in seconds. (n°) = measured in degrees. (pixel) = measured in pixels. (N) = measured in numbers of hits.
